# Multi-Analytical Approach for the Acid-Base, Thermal and Surface Properties Assessment of Waste Biomasses

**DOI:** 10.3390/molecules29235735

**Published:** 2024-12-05

**Authors:** Salvatore Giovanni Michele Raccuia, Emanuele Zanda, Clemente Bretti, Mauro Formica, Eleonora Macedi, Andrea Melchior, Marilena Tolazzi, Martina Sanadar, Davide Lascari, Giovanna De Luca, Anna Irto, Concetta De Stefano, Paola Cardiano, Gabriele Lando

**Affiliations:** 1 Dipartimento di Scienze Chimiche, Biologiche, Farmaceutiche e Ambientali, Università degli Studi di Messina, 98168 Messina, Italy; saraccuia@unime.it (S.G.M.R.); emanuele.zanda@unime.it (E.Z.); cbretti@unime.it (C.B.); giovanna.deluca@unime.it (G.D.L.); cdestefano@unime.it (C.D.S.); glando@unime.it (G.L.); 2 Dipartimento di Scienze Pure e Applicate, Università degli Studi di Urbino “Carlo Bo”, 61029 Urbino, Italy; mauro.formica@uniurb.it (M.F.); eleonora.macedi@uniurb.it (E.M.); 3 Dipartimento Politecnico di Ingegneria e Architettura, Laboratorio di Tecnologie Chimiche, Università di Udine, 33100 Udine, Italy; andrea.melchior@uniud.it (A.M.); marilena.tolazzi@uniud.it (M.T.); martina.sanadar@uniud.it (M.S.); 4 Dipartimento di Fisica e Chimica—Emilio Segré, Università di Palermo, 90128 Palermo, Italy; davide.lascari@unipa.it

**Keywords:** agri-food waste, circular economy, biomass valorisation, biosorbents, biobased material characterisation, acid-base properties

## Abstract

A multi-analytical approach was used to comprehensively characterize the acid-base, thermal, and surface properties of agri-food processing wastes (i.e., original and pre-treated bergamot, grape and olive pomaces). These biomasses, often underutilised and inadequately studied in terms of their physicochemical properties, were investigated under varying ionic strength conditions at *t* = 25 °C. This investigation uniquely integrates multiple advanced techniques: Brunauer–Emmett–Teller porosimetry, Scanning Electron Microscopy, Thermogravimetric Analysis coupled with Fourier Transform Infrared Spectroscopy, Differential Scanning Calorimetry, Attenuated Total Reflectance Fourier-Transform Infrared, and potentiometry to provide a holistic understanding of these biomasses potential for environmental remediation. The modelling of ionic strength-dependent acid-base behaviour, established using an extended Debye–Hückel-type equation, revealed the dominant role of carboxylic groups as active sites across all pomace types, although with variations in abundances across the different samples. Additionally, morphological analysis highlighted the presence of irregularly shaped particles, heterogeneous size distributions, and distinct thermal stability trends, with grape pomace exhibiting the highest mass loss. These findings underscore the significant potential of these biomasses for the remediation of cationic pollutants from natural waters. Moreover, this comprehensive characterisation not only advances the understanding of agri-food waste valorisation but also provides a robust framework for designing targeted strategies in environmental applications.

## 1. Introduction

The agri-food industry generates approximately 1.3 billion tons of waste by-products annually [1], posing potential environmental, economic and social challenges if not adequately managed, treated and/or reused [2]. In fact, the global productions every year of grape and olive pomaces are around 10.5–13.1 [3] and 15.6 million tons [4], respectively. Citrus fruit manufacturing generates approximately 15 million tons of by-products worldwide annually [5]. Among them, orange, lemon, mandarins, grapefruit are the most processed citruses. On the contrary, bergamot, despite it being produced only in some areas of Southern Italy, is a scarcely studied fruit, industrially treated for the extraction of juices and essential oils, and globally exported for nutraceutical, cosmetic and perfumery applications [6]. Its industrial supply chain produces around 55,000 tons of bergamot pomace every year. Among the objectives outlined in the 2030 Agenda [7], the adoption of circular economy [8] emerges as a viable solution for sustainable agricultural waste management and the pursuit of the zero-waste paradigm. Circular economy is a production and consumption model centred around the 4-R strategy—Reduction, Reuse, Recycle and Recovery [9]—aimed at transforming existing products and wastes into new secondary *raw* materials, thus prolonging their life cycle.

In this context, various agri-food by-products have already been reused as soil amendments and biobased fertilisers, for animal feed, in compost production, as heating fuel or as substitutes for fossil fuels [10,11]. In addition, owing to their content of bioactive compounds, such as polyphenols, flavonoids and flavanols [11], vitamins, proteins, fibres, tannins [12], fatty acids, and natural pigments, and their secondary metabolites (e.g., carotenoids, anthocyanins, chlorophylls, betalains) [13], which exhibit several beneficial effects, agricultural biomasses have been employed in the food, pharmaceutical and cosmetic industries [14]. In recent years, various fruit [15] and vegetable peels, seeds, and pomaces [11], as well as nut and egg shells and marine algal wastes [15], have been used and chemically, physically and/or (hydro)thermally modified [16,17] to produce new multifunctional materials capable of interacting with both organic (pesticides, dyes, pharmaceuticals, drugs of abuse, hormones, components of personal care products, Persistent Organic Pollutants) [18] and inorganic (metal cations, anions) target contaminants [19], facilitating their potential selective removal from the environment through adsorption mechanisms [6].

In recent years, the employment of bio-based materials has been proven as a potentially promising answer to the water and wastewater purification issue [20,21]. The use of agri-food wastes as potential recycling biosorbents follows the Green Chemistry principles of “less hazardous chemical synthesis” and “inherently safer chemistry for accident prevention” [22]. It allows for highly efficient, environmentally friendly and economically convenient pollutant sequestration, with reduced sludge production [23], as a valuable alternative to the traditional usage of commercial adsorbents (i.e., silica, activated carbon, chitosan, resins, zeolite) [24]. Agricultural matrices are featured by a porous structure made of biocompatible, biodegradable and non-toxic biopolymers such as lignin, cellulose, hemicellulose and pectins. They also contain carboxylic acids such as D-galacturonic and uronic acids, as well as amino acids, phosphates and micronutrients [1]. These compounds are mainly rich in oxygen-containing functional groups, like hydroxyl and carboxylic sites, which are typically responsible for the biomass adsorption capability [6,25]. Some authors also identified the presence of phosphate and amino groups in citrus and algal waste samples [26,27]. Biosorption occurs when the acidic sites, in their deprotonated forms at suitable pH values, exchange H^+^ with metal cations (M^n+^), for example, in an aqueous solution [25]. The contaminant(s) can be desorbed using strong mineral acids and/or chelating agents, and the sorbent reusability should be then properly checked [28].

In the literature, numerous papers have already reported on the characterisation of native and chemically pre-treated materials derived from agricultural wastes [6,25,27,29,30,31,32,33,34,35,36,37,38,39,40,41,42,43,44,45,46,47,48,49,50,51,52,53,54,55,56,57,58,59,60,61,62]. Chemical reagents, frequently employed to modify bio-based sorbents and improve their interacting capacities towards pollutants, are inorganic (HCl, HNO_3_, H_2_SO_4_, H_3_PO_4_) or organic (tartaric, oxalic, citric) acids, bases (NaOH, Ca(OH)_2_), aldehydes (formaldehyde), alcohols (methanol, 2-propanol), or oxidising agents (H_2_O_2_) [6,25,51,55,63]. Unfortunately, to the best of the authors’ knowledge, no existing single studies have comprehensively focused on the identification of the biomass functional groups potentially capable of interacting with metal ions M^n+^, on the active site concentrations and acid-base behaviour, or on the thermal and surface properties and particle sizes of the new bio-based materials, essential factors governing the material adsorption capability towards the target contaminants [64]. Furthermore, typical conditions employed to carry out the investigations lack an ionic medium or include only a single ionic strength (e.g., *I* = 0.10 mol dm^−3^) [6,25,27,44].

In the present work, a comprehensive multi-analytical approach was employed for the characterisation of original (*dry*) and pre-treated bergamot (BP), grape (GP) and olive (OP) pomaces, with a special focus on their acid-base properties. This serves as a valuable tool for the development of strategies aimed at the remediation of cationic contaminants like metal cations from aqueous solutions or real natural waters. Specifically, the Brunauer–Emmett–Teller (BET) and Scanning Electron Microscopy (SEM) techniques were employed to assess surface area and particle size distribution. Thermogravimetric Analysis coupled with Fourier Transform Infrared Spectroscopy (TG-IR), Differential Scanning Calorimetry (DSC) and Attenuated Total Reflectance Fourier-Transform Infrared (ATR FT-IR) spectroscopy were used to investigate thermal properties and confirm the presence of functional groups potentially responsible for M^n+^ adsorption, respectively. In addition, the acid-base properties of the pomace active sites were investigated by potentiometry for the first time at different ionic strengths (e.g., *I* = 0.10–1.00 mol dm^−3^) in NaNO_3(aq)_ and at *t* = 25 °C. The obtained results were also compared with the data obtained using the classic volumetric method with calcium acetate [65]. For some samples, the point of zero charge was also determined [66]. The dependence of protonation constants on ionic strength was modelled using an extended Debye–Hückel-type equation, providing valuable insights into thermodynamic parameters essential for the calculation of equilibrium constants at various *I*/mol dm^−3^ conditions, including the ones typical of environmental relevant fluids.

## 2. Results and Discussion

### 2.1. Moisture Content and Pre-Treatments

Details on the for the agri-food waste sample supply and on the experimental procedures employed for biomass desiccation and pre-treatments using deionised water and nitric acid solutions are reported in the Section 3. The pristine (*raw*) BP and GP samples dehydration resulted in a weight loss related to the removal of water and volatile compounds. The mass loss was approximately 85% for bergamot [6] and 60% for grape pomaces. These findings align well with reported moisture content values for citrus waste (73–85%) [19,67] and grape waste (40–75%) [68,69], which vary depending on factors such as fruit variety, ripeness degree [70], and pressing conditions [71]. As for OP, which was already supplied in the dried form, various authors have reported moisture ranging from 25% to 55% for pomaces from different olive cultivars [12,69,72,73].

*Dry* BP, GP and OP samples underwent additional pre-treatments with H_2_O and HNO_3_ 0.1019 mol dm^−3^ solutions following the procedure outlined in the Section 3. Further details are listed in Table S1. These chemical treatments aimed to assess the potential enhancement of the agri-food biomass sorption ability towards cationic pollutants like metal cations for future applications in real natural waters. Furthermore, pre-treatment with water was selected in order to eliminate the water-soluble molecules, whilst nitric acid was employed, aimed at protonating the available functional groups, thus removing the alkaline (Na^+^, K^+^) and alkaline-earth (Ca^2+^) metal cations, as well as favouring the disclosing of the biomass physical structure, increasing the availability of surface-binding sites [49,63].

As already observed in a previous paper focusing on bergamot pomace [6], the pre-treated samples underwent additional weight loss. Specifically, the weight loss was found to be 52% and 60% for BP H_2_O and BP HNO_3_, respectively (Table S1). In the case of grape and olive waste samples, mass losses of 38–39% and 21–24% were observed for water and nitric acid pre-treated GP and OP, respectively. To the best of the authors’ knowledge, no other information regarding biomass weight loss percentages after chemical pre-treatments is available in the literature.

### 2.2. ATR FT-IR Characterisation of Pomaces

The ATR FT-IR analyses of both native (*dry*) and pre-treated BP samples had already been conducted and discussed in a previous paper [6]. The signals observed in the bergamot pomaces and their corresponding attributions to specific functional group vibrations exhibited numerous similarities with those previously reported for original citrus (e.g., lemon, orange, pomelo, and grapefruit) peels [40,49,50,54,55,59] and wastes [46,58] sourced from Spanish, Floridian, and Taiwanese local market or industries. Additionally, analogies were identified with data obtained from samples subjected to pre-treatment with diluted solutions of HCl [50], oxalic acid, and NaOH [54].

**Figure 1:**
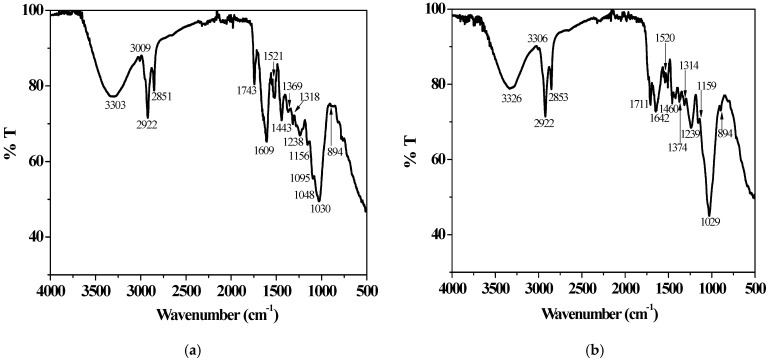
ATR FT-IR spectra recorded for the grape (**a**) and olive (**b**) pomace samples after pre-treatment with deionised water.

The spectra recorded for the *dry*, H_2_O- and HNO_3_-pre-treated grape pomaces (Figure 1a and Figure S1a,b) showed profiles comparable to those reported in the literature for red grape wastes with various granularities supplied by winemaking companies from Brazil [34], China [52,62], and Romania [48,70]. Similarities were also observed in the case of GP samples chemically treated with 1.5% H_2_SO_4_ and 2% NaOH solutions [52]. The presence of broad bands at 3310–3270 cm^−1^ is possibly ascribable to the vibrations of hydroxyl groups in phenolic and polysaccharide structures, such as cellulose and hemicellulose [70]. The small signal around 3010 cm^−1^ could be related to the aromatic C–H stretching in polyphenolic compounds, while the ones at 2921 cm^−1^ and 2851 cm^−1^ may be attributed to the methylene groups of polysaccharides and of triglycerides from grape seed oil [74,75]. The band at 1743 cm^−1^ may correspond to the stretching band of non-ionic C=O group, suggesting the presence of uronic acid [34]. According to the literature information, the signals at 1609 cm^−1^ could be assigned to aromatic C–H bonds [62] and ionic carboxylic C=O stretching vibrations [48], while the ones at 1528–1524 cm^−1^ could be assigned to the C=C stretching bands, typical of aliphatic and aromatic molecules [62]. Peaks at 1441–1443 cm^−1^ and 1368–1378 cm^−1^ could be attributed to the antisymmetric and symmetric in-plane bending of CH_3_ groups, respectively [34]. Peaks at 1230–1250 cm^−1^, 1136–1156 cm^−1^, and around 1030–1050 cm^−1^ are generally imputable to the aromatic C–O–C stretching vibrations owing to the presence of cellulose and hemicellulose [62], as well as to the C–O stretching bands, due to the presence of pyranose rings. The signals at 903 cm^−1^, 894 cm^−1^, 881 cm^−1^ may suggest the presence of β-glycosidic [52], β-D-pyranose and phenolic compounds in the grape pomace matrix [34].

The spectrum of *dry* olive pomace (Figure S1c) closely resembles those reported in the literature for native biomass samples supplied by Italian, Spanish and Turkish olive oil production companies [25,30,44,47]. On the contrary, ATR FT-IR profiles recorded for olive pomaces pre-treated with 50% H_3_PO_4_ and 1 mol dm^−3^ H_2_O_2_ solutions are quite different, likely due to partial and significant chemical modifications, respectively, compared to the original biomass [25]. Some similarities were instead observed with the ATR FT- IR findings for grape pomaces, such as the attributions of the broad band at 3316–3334 cm^−1^, the small signal at around 3010 cm^−1^ and the peaks at 1711 cm^−1^ and 1639–1652 cm^−1^. The signals at 2922–2928 and 2853 cm^−1^ could be ascribable to the –H vibrations of CH_3_, CH_2_ and CH groups in saturated fatty acids. The aromatic ring C–C stretching of polyphenols could be highlighted by the presence of peaks at 1512–1526 cm^−1^ [44]. The main difference between the spectra of *dry* (Figure S1c) and pre-treated pomaces (Figure 1b and Figure S1d) primarily lies in the relative intensity ratios among signals in the wavenumber range of 1512–1710 cm^−1^. The increased absorbance at 1710 cm^−1^ observed in the OP sample pre-treated with HNO_3_ suggests a possible oxidation process occurring during acidic pre-treatment on the polyphenol fraction, leading to the formation of carboxylic acids. Peaks at 1460 and 1310–1320 cm^−1^ may be imputable to cellulose and lignin vibration bands, while the CH_2_ bending mode of cellulose could be observed at 1360–1377 cm^−1^. The bands at 1019–1029 cm^−1^ and around 1159 cm^−1^ may be attributed to C–O and C–C stretching, as well as to the O–C–O symmetric stretching of the glycosidic bond, respectively, in pectin and cellulose. Pectin C–O stretching may be observed at 1235–1239 cm^−1^ [47]. The signal at 894 cm^−1^ could be assigned to the C–O–C stretching in cellulose [6].

### 2.3. Pomace Acid-Base Properties and Active Site Concentrations

#### 2.3.1. Equilibrium Constants

The acid-base behaviour of *dry* and pre-treated bergamot, grape and olive pomaces was potentiometrically studied at *I* = 0.10–1.00 mol dm^−3^ in NaNO_3(aq)_ and at *t* = 25 °C across the pH range of 2.0–11.0. Throughout the titrations, the initially transparent solutions containing the orange-brown solid samples at pH ~ 2.0 turned yellow at pH ≥ 7.0, maintaining this coloration until the conclusion of each experiment.

The BSTAC computer program [76], discussed in the Section 3, capable of efficiently handling data collected at different *I*/mol dm^−3^ values, was employed to determine the acidic (protonation) constants at infinite dilution and the corresponding *C* parameter for the dependence on ionic strength (extended Debye-Hückel type equation, Equation (4)). At first, the entire pH range of the investigation was considered for the data elaboration. For some BP, GP and OP samples, two equilibrium constants, possibly corresponding to two types of active sites, namely hydroxyl (log*K*^H^_1_ = 10.5–10.8) [27] and carboxylic groups linked to aromatic moieties (log*K*^H^_2_ ~ 3.0) or aliphatic (log*K*^H^_2_ ~ 5.0) chains [25], were determined. However, in some cases, the calculations were unsuccessful, and the obtained *C* values varied considerably among the different pomaces.

Few papers have been published on the acid-base properties of agri-food biomasses, and, to the best of authors knowledge, no data have been reported regarding the acidic behaviour of grape pomace active sites. Pagnanelli et al. [44] and Martin-Lara and co-workers [25] potentiometrically investigated the acid-base properties of native olive pomaces and pre-treated samples in the absence of ionic medium with phosphoric acid and hydrogen peroxide. The authors, using discrete and continuous models, considered the main presence of two weakly monoprotic acidic active groups [25,44] in the solid biomasses. For the three samples, the first approach led them to obtain values of log*K*_1_^H^ = 8.60–8.88 and log*K*_2_^H^ = 3.33–4.39, and the second one led them to values of log*K*_1_^H^ = 8.77–9.50 and log*K*_2_^H^ = 3.60–5.00. The first acidic constants are around 1.5–2.0 orders of magnitude lower than the data obtained here using the BSTAC computer program and abovementioned. On the contrary, the second equilibrium constants are more like the BP, GP and OP experimental ones. Schiewer et al. [27] reported the results of a potentiometric investigation on HCl protonated orange peels. A continuous p*K*_a_ spectrum [77] suggested the existence of four acidic sites related to phenolic, phosphate (two log*K*^H^) and carboxylate active sites with log*K*^H^ = 10.7, 8.4, 6.4, 3.8. Pipíška and co-workers [26] predicted the presence of -COOH, -PO_3_H_2_, -NH_2_, -OH groups in freshwater moss *V. dubyana* with acidic constants in the ranges 2.34–6.25, 6.36–8.72, 10.7–11.5, 8.36–12.0, respectively. In both cases, the biomass active sites are predominantly carboxylic groups. The few literature data primarily focused on the pH range 2.0–11.0, and detailed information on experimental observations during the titrations, such as changes in suspension and solution colour, were not typically reported.

Based on all these findings and discussions, and for trying to ulteriorly elucidate the biomass acid-base behaviour, aliquots of the filtered solutions, obtained after potentiometric measurements on bergamot pomace samples pre-treated with H_2_O, were back-titrated using HNO_3(aq)_. The starting yellow solutions at pH ~ 11.0 became transparent again at pH ~ 7.0 and up to ~ 2.0. The corresponding data were elaborated and values of log*K*^H^_1_ = 10.5–10.8 were determined at 11.0 ≤ pH ≤ 7.0. These results could suggest that the hydroxyl groups, likely present in protonated form in the BP samples at acidic pH, and exhibiting acid-base behaviour at alkaline values, could solubilise in aqueous solutions at pH ≥ 7.0, likely contributing to the yellow coloration. The refinement of protonation constants during the back-titration was also checked at 7.0 ≤ pH ≤ 2.0, with the aim of testing the possible presence in solution of COOH active sites [44,78]. The calculations were not successful, suggesting that the species containing these groups may not solubilise in aqueous solution during the initial measurements, perhaps remaining on the biomass solid sample.

Based on these considerations, the final acid-base data treatment was carried out at pH = 2.0–7.0. For each pomace sample, the BSTAC computer programme allowed the determination of one protonation constant (log^T^*K*^H^) and the empirical *C* parameter at infinite dilution reported in Table 1. Using the extended Debye–Hückel-type equation (Equation (4)), it was also possible to refine the values of acidic constants (Table S2) at various ionic strength conditions within the experimental *I*/mol dm^−3^ range of investigation. Noteworthy, each BP, GP and OP active group was arbitrarily indicated as a mono-negatively charged site throughout the calculations and text.

#### 2.3.2. Active Site Concentrations

For all the biomasses, the concentration of -COOH groups in meq g^−1^ was also determined by means of the BSTAC computer program as well as the results reported in Table 1. The *dry* samples displayed a higher concentration of active sites compared to those treated with H_2_O and HNO_3(aq)_. Accordingly, Pagnanelli et al. [44], using the Gran’s method [79], predicted values of 0.67 and 0.32 meq g^−1^ for native olive pomace and after the pre-treatment with 0.10 mol dm^−3^ NaOH solution. For these samples, the same author determined active site amounts of 0.28 and 0.24 meq g^−1^, respectively, at 2.0 ≤ pH ≤ 8.0. Only for the original OP sample, carboxylic group titrations were also performed using a 0.05 mol dm^−3^ NaHCO_3_ solution, and a concentration of 0.05 meq g^−1^ was calculated. This value is much lower than the literature predicted and elaborated datajust discussed, as well as lower than the ones reported in Table 1 for olive pomace samples. Martin-Lara and co-workers [25], for original and H_3_PO_4_ pre-treated OP samples, experimentally obtained -COOH site concentrations 0.35 and 0.17 meq g^−1^ using the discrete model and 0.41 and 0.15 meq g^−1^ with the continuous one. Although these literature results have been obtained by also considering the hydroxyl groups as further active sites (determinable at alkaline pHs), the abovementioned data can be considered in quite good agreement with the ones here determined for *dry* OP (0.43 meq g^−1^), as well as those for the H_2_O (0.21 meq g^−1^) and HNO_3(aq)_ (0.15 meq g^−1^) pre-treated samples. To the best of the authors’ knowledge, no values of active site concentration are reported in the literature for grape pomaces.

In Table 1, it is also possible to observe that the bergamot pomace subjected to pre-treatment with deionised water exhibits fivefold higher (1.10 meq g^−1^) acidic active group amounts compared to those calculated for the corresponding grape (0.23 meq g^−1^) and olive (0.21 meq g^−1^) waste samples. Schiewer et al. [27] and Pipíška and co-workers [26] determined values of carboxylic site concentrations of 0.39 meq g^−1^ and 0.90 meq g^−1^ in HCl-protonated orange peels and freshwater moss *V. dubyana*, respectively, suggesting that bergamot pomace is richer in -COOH groups than other agri-food and natural biomasses.

To further verify the robustness and accuracy of the biomass site amounts in Table 1, as examples, two additional experimental approaches were employed on grape and olive pomace samples pre-treated with water. The first approach involved a larger biomass quantity (around 250 mg) and performing potentiometric titrations at *I* = 0.10 mol dm^−3^ in NaNO_3(aq)_. The data analysis in the pH range 2.0–7.0 also led to the determination of log*K*^H^ = 4.14 ± 0.03 and 4.80 ± 0.01 for GP and OP samples, respectively. These values are in quite good accordance with the data calculated using Equation (3) at the same ionic strength reported in Table S2. The active site concentrations were 1.20 ± 0.01 meq g^−1^ for GP H_2_O and 1.51 ± 0.02 meq g^−1^ for OP H_2_O, corresponding to 0.24 and 0.30 meq g^−1^, respectively, per 50 mg of biomass. These data well fit with the ones reported in Table 1 of 0.23 and 0.21 meq g^−1^ for grape and olive samples pre-treated with deionised water.

The second approach utilised the classic volumetric method with calcium acetate on around 50 mg of the same samples [65]. Following the experimental procedure reported in the Section 3, and considering the titrant volumes (TVs) at equivalent points for pomaces and blank samples, the titrant concentration, and the biomass weight, Equation (2) [80] was employed to calculate the carboxylic site concentration in the analyzed samples. For both the biomasses, an amount of 0.24 meq g^−1^ was determined, further confirming the data reported in Table 1. Overall, these additional experimental approaches validate the accuracy and robustness of the active site concentrations reported in Table 1.

### 2.4. Point of Zero Charge (PZC)

The point of zero charge (PZC) indicates the pH value at which the biosorbent has a net surface charge of zero, due to an equal amount of positive and negative charges [33]. This is dependent on the acid-base properties of the active sites [26], as discussed in the Section 3. The PZC is also defined as the pH in which material anion and cation exchange capacities are equivalent [81].

**Figure 2:**
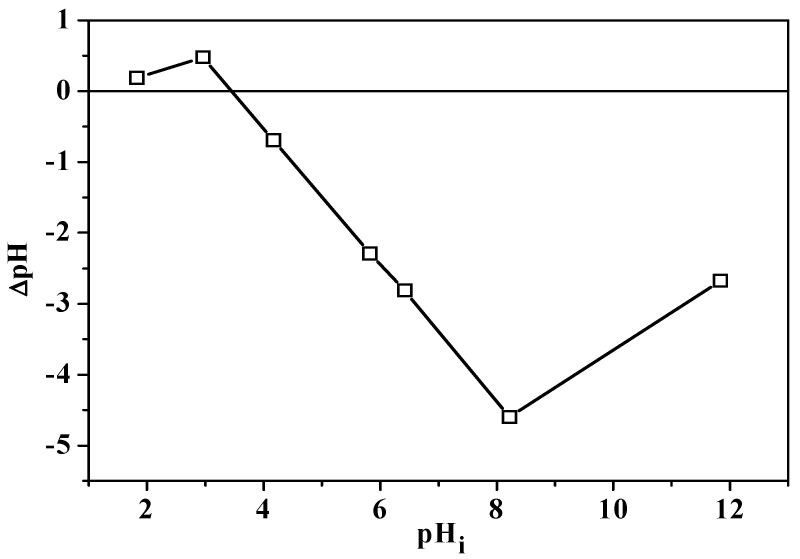
ΔpH vs. pH_i_ plot for the PZC determination for the BP H_2_O sample.

The point of zero charge was investigated, following the experimental procedure reported in the Section 3 as well, for three selected samples, bergamot and grape pomaces pre-treated with water and *dry* OP, at *I* = 0.10 mol dm^−3^ in NaNO_3(aq)_ and at *t* = 25 °C. The experimental plots of ΔpH against pH_i_ and the intersection point with the x-axis are presented in Figure 2 and Figure S2. The PZCs determined for BP H_2_O, GP H_2_O and *dry* OP are 3.46, 3.96, and 5.82, respectively.

The PZC value for bergamot pomace (Figure 2) is quite similar to the value of 3.95 reported by Šehović and colleagues [51] at the same temperature and ionic strength in NaNO_3(aq)_ for native lemon peel. However, it is lower than the point of zero charge of 5.67 determined by the same author for a sample doubly pre-treated with 0.25 mol dm^−3^ HNO_3_ and 0.10 mol dm^−3^ NaOH solutions. Thirumavalavan and coworkers [55] and Fernández-López et al. [39] obtained PZC values of 4.65 and 4.20 for original lemon peels and citrus waste, in the absence of an ionic medium and at *I* = 0.03 mol dm^−3^ in KNO_3(aq)_, respectively, at *t* = 25 °C. At an analogous temperature and in deionised water, Stadnik’s group [53] assessed point of zero charge values of 5.80 and 4.50 for different varieties of orange pomaces, and a 5.50 for orange peel. The PZCs here determined for GP H_2_O (Figure S2a) can be compared with the values of 3.00 and 4.60 reported by de Oliveira et al. [37] and Gonzales-Condori and coworkers [82] at *I* = 0.10 mol dm^−3^ in NaNO_3(aq)_ and at the same temperature for grape pomace and seed waste, respectively. Fernández-López’s group [39] also reported a point of zero charge of 5.40 for olive mill residue, which is quite similar to the value here found for *dry* OP (Figure S2b). Ali et al. [30] determined a PZC of 9.00 for olive pomace at *I* = 0.01 mol dm^−3^ in NaNO_3(aq)_ and at *t* = 25 °C. At an analogous temperature and in the same supporting electrolyte, Alrowais and colleagues [83] obtained a PZC of 7.70 for olive fruit waste at *I* = 0.45 mol dm^−3^, while Uzunkavak and Özdemir [57] reported a value of 6.20 for olive pomace at *I* = 0.01 mol dm^−3^ in NaCl_(aq)_ solution and at *t* = 30 °C.

### 2.5. Surface Area

The Brunauer–Emmett–Teller (BET) method was employed to investigate the surface area of the different biomass samples. As observable in Table 2, the values obtained for the *dry* and pre-treated bergamot, grape and olive pomaces are in quite good agreement with the data (0.41, 0.54 m^2^ g^−1^) reported by Villen-Guzman et al. [58] for *raw* lemon peels and samples pre-treated with NaOH, respectively. On the contrary, higher olive waste and citrus peel surface areas were determined by various authors [36,38,39,55,60]. In particular, Thirumavalavan and co-workers [55] measured values of 2.00 and 1.27 m^2^ g^−1^ for orange and lemon peels, El Malti et al. [38] 1.90 m^2^ g^−1^ for *Citrus sinensis* peel, and Chao and colleagues [36] 1.62 m^2^ g^−1^ for pomelo peel. Fernández-López et al. [39] determined surface areas of 3.73 and 1.80 m^2^ g^−1^ analyzing olive mill residue and citrus waste. Ye and colleagues [60] investigated insoluble dietary fibres gained from citrus pomace and obtained a 0.94 m^2^ g^−1^ value.

### 2.6. SEM

**Figure 3:**
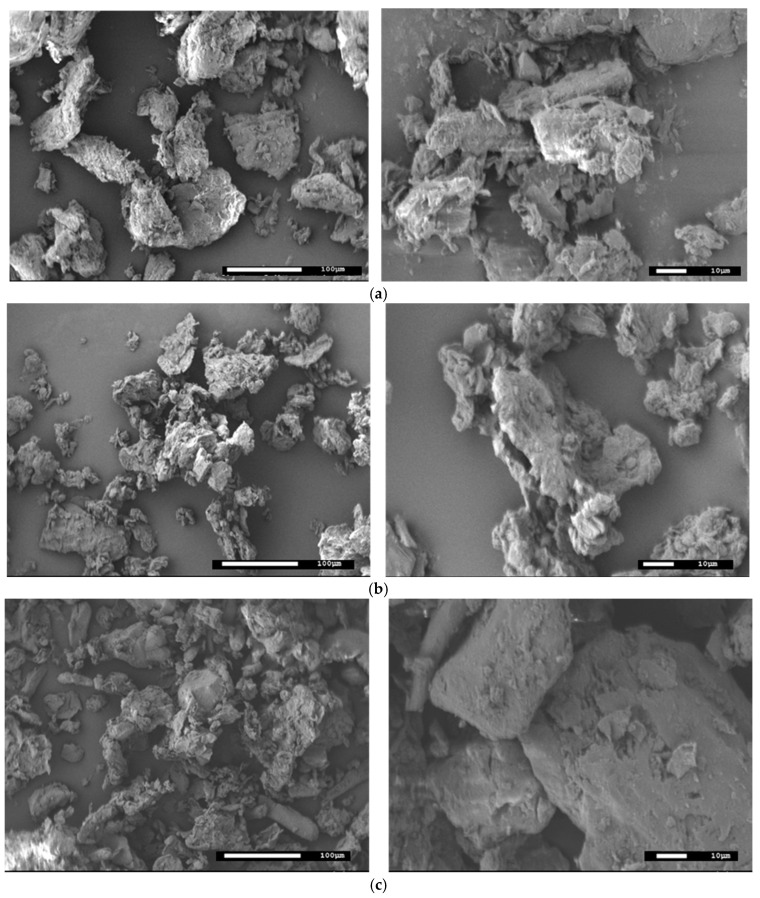
SEM images of H_2_O-pre-treated BP (**a**), GP (**b**) and OP (**c**) samples. Left ×250, right ×1000 magnification.

In Figure 3, the SEM images of H_2_O-pre-treated adsorbent materials are shown. The three biomasses consist of particles with irregular shapes and varying size distributions, with lengths up to approximatively 100 μm. Since the morphology of the *dry* and HNO_3_ pre-treated samples did not exhibit significant differences, they are not included in this figure. In the literature, various authors have observed heterogeneous surfaces and irregular geometries in both original and NaOH-modified citrus (e.g., lemon, orange) [38,39,51,54,55,58,59], grape pomaces [43], and olive mill wastes [31,39]. In olive pomace, Ali et al. [30] individuated porous structures with surface cavities possibly capable of interacting with contaminants. Only a few of these studies reported data on particle size distribution. Fernández-López et al. [39] identified packed and porous patterns of around 70 μm in both olive mill residue and citrus waste, and Zannini [61] and colleagues measured sizes of 50 μm in citrus pectins, while Melia and coworkers reported a size of 100 μm in grape pomaces. Titone and colleagues [56] observed structures with irregular shapes and varying sizes in the interval between a few microns and 200 µm in grinded grape pomace samples. Kaya and coworkers [41] identified particles both smaller than 1 μm and those larger than 100 μm in olive pomaces. Villen-Guzman’s group [58,59] observed sizes of approximately 10–20 μm in both unmodified lemon peels and NaOH pre-treated samples. They also noted variations in morphological and surface structure after alkaline chemical treatment, likely due to increased adsorption capacity.

### 2.7. TG-IR

The TG-IR analyses were performed on *dry* and pre-treated adsorbent materials in the temperature interval 30–800 °C at a heating rate of 20 °C min^−1^ under a N_2_ atmosphere (70 cm^3^ min^−1^).

**Figure 4:**
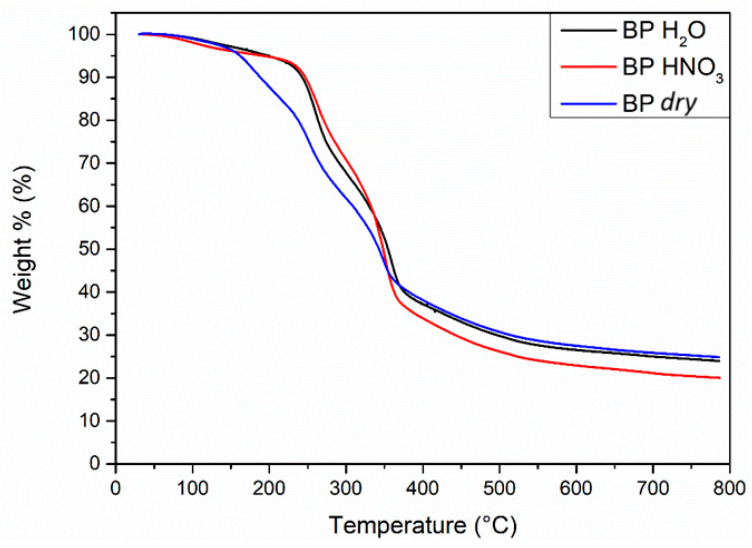
Superimposition of TGA thermograms recorded for BP samples.

The TGA thermograms for all samples always showed several not-well-resolved weight loss steps. Figure 4 depicts the thermal behaviour of bergamot pomace samples. The decomposition of *dry* BP (blue line in Figure 4) showed the occurrence of five weight loss steps in the temperature ranges 30–120 °C (with a weight loss of 1.7%), 120–210 °C (12%), 210–300 °C (24%), 300–410 °C (25%) and 410–600 °C (10%) (Table 3). After the last step, the weight stabilised around 25%. Upon the pre-treatment with H_2_O and HNO_3_, the BP samples (black and red lines for BP H_2_O and BP HNO_3_, respectively, in Figure 4) displayed the occurrence of four weight loss steps. The behaviours of the two samples are quite similar to each other, with 4.7–4.9% and 28.7–23.5% weight losses in the first two intervals (*t* = 30–190 °C and 190–300 °C), as well as 30% and 38% (temperature range 300–412 °C) for BP H_2_O and BP HNO_3_ in the third step, respectively. Lastly, (*t* = 412–610 °C or 412–560 °C, for the two samples, respectively) a weight loss of 9.8 and 8.9% was observed for the two samples, respectively. After the last step, the weight stabilised at 24% and 20% for BP H_2_O and BP HNO_3_, respectively (Table 3).

The olive and grape pomaces showed similar thermal behaviour with respect to that for BP, with between five and six weight loss steps (Figures S3 and S4 in the Supplementary Material). Temperature intervals and corresponding weight loss percentages for all the samples are reported in Table 3. In all cases, the pre-treated biomasses showed thermograms quite similar to each other, and different from the *dry* samples.

In the literature, Zannini et al. [61] reported the TG profile recorded for a lignocellulosic residue extracted from citrus pomace represented by three phases of weight loss. The first step, occurring at *t* = 50–150 °C, was attributed to the evaporation of water absorbed in the sample and to the low-molecular-weight polysaccharide decomposition. The one observed between 150 and 400 °C was assigned to lignin, hemicellulose and pectin degradation, whilst the phase noticed at *t* = 400–600 °C was attributed to lignin polymeric compound decomposition. Quite similar attributions were suggested by Bennini and colleagues [35], who investigated the thermal profile of *raw*, exhausted and de-oiled olive pomace samples. The three weight loss steps were determined at *t* = 0–150 °C (7–15%), 150–400 °C (65–75%) and 400–800 °C (20–30%). Madadian and coworkers [42] analyzed the thermal decomposition of grape pomace powder samples. They identified a first step at *t* = 150–200 °C (5%) assigned to moisture loss, a second one at *t* = 80–320 °C (67%) related to the organic compounds devolatilisation with subsequent char formation, and a third step at *t* = 400 °C (23%) attributable to char combustion.

**Figure 5:**
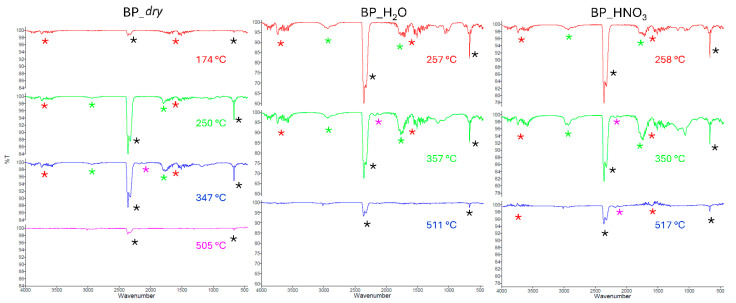
FT-IR of gasses evolved during the decomposition of BP samples. The temperatures where the spectra were recorded are also reported. CO_2_ (black stars), water (red stars), formaldehyde (green stars), and CO (magenta stars).

The FT-IR analysis of the evolved gas during the TGA of bergamot pomaces is reported in Figure 5, while analyses for OP and GP samples are shown in Figures S5 and S6. All the samples evolved with CO_2_ (black stars) and water (red stars), which were retrieved basically in all steps. The mixture of gasses were enriched in formaldehyde (green stars), above *t* = 240 °C, and CO (magenta stars), starting from above *t* = 300 °C.

### 2.8. DSC

The DSC curves of all the samples obtained in the N_2_ atmosphere (30 cm^3^ min^−1^) at a heating rate of 20 °C min^−1^ from *t* = 30 to 400 °C show a series of not-well-resolved thermal events, in line with the decomposition processes observed with the TGA analysis. No melting peaks can be seen, and all thermal events observed in the DSC curves are due to the dehydration and decomposition of the samples and associated with the pronounced weight losses resulting from the TGA. The cooling curves did not show any thermal event.

The DSC signal recorded for the *dry* BP sample (blue curve in Figure S7) is mainly characterised by an endothermic peak at *t* = 160 °C, associated with the loss of water from the sample, and an exothermic event at *t* = 347 °C. BP H_2_O and BP HNO_3_ samples (black and red curves in Figure S7, respectively) showed similar DSC curves, with anticipated dehydration at *t* = 104 and 93 °C, respectively, and similar exothermic peaks at *t* = 356 and 352 °C, respectively.

The DSC curve of the *dry* OP sample (blue curve in Figure S8) is mainly characterised by an endothermic peak at *t* = 83 °C, associated with the loss of water, and an exothermic event at *t* = 338 °C. The DSC profile is in agreement with the one previously observed by Akcicek et al. [29] for olive pomace extract, even if the dehydration temperature is lower in the present case (endothermic dehydration peak: *t* = 83 vs. 147 °C; exothermic peak: *t* = 338 vs. 335 °C). OP H_2_O and OP HNO_3_ samples (black and red curves in Figure S8, respectively) showed a similar dehydration process (*t* = 82 °C), but no exothermic peaks in the 300–350 °C temperature interval.

The DSC curve of the GP-*dry* sample (blue curve in Figure S9) is mainly characterised by an endothermic peak at *t* = 99 °C, associated with the loss of water from the sample, and a sharp endothermic peak at *t* = 273 °C, due to the presence of pectin [84]. A small exothermic peak is present at *t* = 365 °C. GP H_2_O and GP HNO_3_ samples (black and red curves in Figure S9, respectively) showed a similar dehydration process (*t* = 96 °C) and an exothermic peak at *t* = 368 °C, but the pectin endothermic peak disappeared.

## 3. Materials and Methods

### 3.1. Agri-Food Waste Samples and Chemicals

An amount of 5 kg of bergamot pomace (BP) was collected from the *Femminello* and *Fantastico* cultivars between November 2021 and January 2022. The biomass, comprising seeds, pulp and deoiled citrus flavedo after juice and essential oil extraction, was sourced from Capua 1880, a company based in Reggio Calabria, Italy. Additionally, 6 kg of white grape pomace (GP) from the *Carricante* cultivar was supplied by the agricultural company Giuseppe Russo (Passopisciaro, Italy). The solid residue (seeds, peel and pips after pressing the berries for must production) was representative of the September 2023 harvesting season. An amount of 10 kg of olive pomace (OP), sampled in November 2022 from the *Nocellara dell’Etna* cultivar and consisting of seeds and deoiled pulp after olive oil extraction, was provided by the Russo (Passopisciaro, Italy) oil miller, located in Randazzo, Italy. The olive waste was already supplied in dehydrated form.

All the chemicals used in the studies were purchased from Merck (Darmstadt, Germany) and were of the highest purity available, requiring no further purification. A list of all the chemicals used in this study can be found in Table S3.

### 3.2. Pomaces Pre-Treatments

Aliquots of the pristine (*raw*) pomaces were mechanically ground using a grinder, dried in an oven at *t* = 60 °C until constant weight and blended again to form a fine powder. The resulting solids, named *dry* BP and GP, and the ground OP were divided in diverse portions and pre-treated for 6 h at *t* = 30 °C, using deionised water and nitric acid solutions, according to procedures reported in the literature [6]. Briefly, the mixtures were centrifuged at 5000 rot m^−1^ and *t* = 25 °C for 5 min for solid–supernatant separation. The solids were washed with deionised water and centrifuged again, up to a constant (± 0.05 pH units) value of pH between two successive washing cycles. Solids were dried in an oven at *t* = 60 °C until constant weight and eventually mechanically ground up to a fine powder. A list of the pre-treated samples is reported in Table S1.

### 3.3. Analytical Instrumentations and Procedures

#### 3.3.1. Potentiometric Measurements

The acid-base properties of the active sites present on *dry* and pre-treated pomace samples were investigated through automatic potentiometric titrations using Metrohm 809 Titrando (Herisau, Switzerland) plugged to a computer and a combined “sure flow” glass electrode purchased from Orion (8172BNWP model, Greenwich, UK). The Metrohm TiAMO 1.2 software was employed to control titrant delivery, data acquisition and *e.m.f.* stability. The estimated accuracies for *e.m.f*. and titrant volume readings were ±0.15 mV and ±0.003 cm^3^, respectively.

Suspensions of 25 cm^3^ containing approximately 50 or 250 mg of *dry* and pre-treated bergamot, olive and grape pomace, nitric acid (*c*_HNO3_ = 10 mmol dm^−3^) to regulate the pH at ~2.0, and NaNO_3_ at different ionic strengths (*I* = 0.10–1.00 mol dm^−3^) were titrated with standard sodium hydroxide solutions (*c*_NaOH_ = 0.0997, 0.0965 mol dm^−3^) up to pH ~ 11.0 in thermostated cells at *t* = 25.0 ± 0.1 °C. For samples pre-treated with deionised water, the suspensions collected after the potentiometric measurements at the various *I*/mol dm^−3^ values were filtered on filter paper, and 25 cm^3^ of the resulting solutions were back-titrated with standard nitric acid solutions (*c*_HNO3_ = 0.0915 mol dm^−3^) up to pH ~ 2.0. Presaturated N_2(g)_ was bubbled into the suspensions and solutions to remove and hinder the presence of oxygen and carbon dioxide.

A method proposed by Schnitzer and Khan [85] was used for the determination of carboxylic group concentrations [65] in grape and olive pomace samples pre-treated with water. Approximately 50 mg of biomass was treated in a stoppered flask with 10 cm^3^ of a calcium acetate hydrate solution (*c* = 0.5034 mol dm^−3^) in 40 cm^3^ of carbonate-free deionised water, favouring the release of acetic acid (Equation (1)):

(1) $$2\;R-\mathrm{COOH}+{(CH_{3}\mathrm{COO})}_{2}\mathrm{Ca}\;\to {\left(\mathrm{RCOO}\right)}_{2}\mathrm{Ca}+2\;CH_{3}\mathrm{COOH}$$

A blank solution was prepared in a capped flask as well. After stirring at room temperature for 24 h, the suspensions were filtered using paper filters. Then, 25 cm^3^ of each filtrate and blank sample was potentiometrically titrated with a standard 0.1004 mol dm^−3^ ($N_{\mathrm{NaOH}}=$0.1004 eq dm^−3^) NaOH solution using the already mentioned combined “sure flow” Orion glass electrode to measure the pH up to ~11.0. The concentration of carboxylic groups in meq g^−1^ was calculated as follows (Equation (2)) [80]:

(2) $$\mathrm{COOH}\;\mathrm{groups}\;\mathrm{concentration}=\frac{({\mathrm{TV}}_{\mathrm{biomass}}-{\mathrm{TV}}_{\mathrm{blank}})\cdot N_{\mathrm{NaOH}}\cdot 1000\;}{\mathrm{biomass}\;\mathrm{weight}}$$

where

${\mathrm{TV}}_{\mathrm{biomass}}\;\mathrm{and}\;{\mathrm{TV}}_{\mathrm{blank}}$ are the titre values, namely the titrant volumes at an equivalent point, for biomass and blank samples, respectively;
$N_{\mathrm{NaOH}}$ is the base concentration on the normality scale (eq dm^−3^), corresponding in this case to the molar one ($c_{\mathrm{NaOH}}$, mol dm^−3^);
biomass weight is the mass in mg of the pre-treated grape and olive pomace samples.

The point of zero charge (PZC) was determined for selected BP, GP and OP samples. For this purpose, 25 cm^3^ aqueous solutions containing HNO_3(aq)_ (*c*_HNO3_ = 0.0997 mol dm^−3^) or NaOH_(aq)_ (*c*_NaOH_ = 0.1002 mol dm^−3^) were prepared to adjust the initial pH (pH_i_) between 2.0 and 11.0. NaNO_3_ was added to regulate the ionic strength (e.g., *I* = 0.10 mol dm^−3^). Aliquots of approximately 300 mg of *dry* olive pomace, bergamot or grape biomass pre-treated with water were put in contact with the solutions for 6 h at *t* = 25 °C. The suspensions were then centrifuged for 15 min at the same temperature and 6000 rot m^−1^ for the solid–liquid phase separation. Then, the supernatants’ final pHs (pH_f_) were measured. The solution pH values were checked using a Metrohm 713 pH meter equipped with a combined glass electrode (6.0233.100 model). The pH variation (ΔpH) was calculated by means of Equation (3):

(3) $$\Delta \mathrm{pH}={\mathrm{pH}}_{f}-{\mathrm{pH}}_{i}$$

The variable changes were plotted against pH_i_ and the PZC was determined as the intersection point with the x-axis [33].

#### 3.3.2. ATR FT-IR

Attenuated total reflectance (ATR) in Fourier transform (FT) Infrared (IR) analyses was determined in the wavenumber range 400–4000 cm^−1^ on bergamot, grape and olive pomace samples, previously ground in an agate mortar to achieve a powder texture. A Nicolet Thermo Scientific FT-IR spectrometer (iS50 model, Waltham, MA, USA) equipped with an ATR diamond window module was employed. For each biomass sample, 32 scans were accumulated with a spectral resolution of 4 cm^−1^.

#### 3.3.3. BET

Surface areas of the samples were measured according to the Brunauer–Emmett–Teller (BET) equation by N_2_ adsorption isotherms at *t* = 196 °C. The measurements were carried out in a Tristar 3000 gas adsorption analyzer (Micromeritics, Norcross, GA, USA). Prior to the adsorption measurements, the samples were degassed at *t* = 80 °C for 6 h.

#### 3.3.4. SEM

The microstructure of the *dry* and pre-treated biomasses was assessed by field-emission electron microscopy (FESEM), JEOL model JSM-7610F Plus (JEOL Ltd. Tokyo, Japan), using a 5 or 15 kV voltage and a working distance of 15 mm. Samples were previously coated with 5 nm layer of gold to increase conductivity.

#### 3.3.5. TG-IR

Thermogravimetric (TG) analyses were performed between *t* = 30 and 800 °C at 20 °C min^−1^ on bergamot, grape and olive pomace samples under a N_2_ atmosphere (gas flow 70 cm^3^ min^−1^, samples between 6 and 20 mg), by using ceramic sample pans. The evolved gasses were simultaneously analyzed by FT-IR. Measurements were carried out on a PerkinElmer TGA 4000 ThermoGravimetric Analyzer (Waltham, MA, USA) interface, through a transfer line (TL8000e EGA) operating at *t* = 270 °C, with an FT-IR Spectrum Two (S Two) Spectrometer equipped with a gas cell, to measure the percentage weight loss of the sample and, at the same time, identify the evolved gas.

#### 3.3.6. DSC

Differential Scanning Calorimetry (DSC) analyses were performed between *t* = 30 and 400 °C (and back) at 15 °C min^−1^ on BP, GP and OP samples under a N_2_ atmosphere (gas flow 30 cm^3^ min^−1^, samples between 5 and 8 mg), by using aluminum sample pans with lids. Measurements were carried out on a PerkinElmer DSC 6000 Differential Scanning Calorimeter.

### 3.4. Modelling for the Dependence on Ionic Strength of Protonation Constants

The dependence on ionic strength of the acidic (protonation) constants determined for the active sites present on the pomace samples was modelled, for the first time, using the extended Debye–Hückel-type equation reported in Equation (4):

(4) $$\log K=\log TK-z^{*}\cdot \mathrm{DH}+C\cdot I$$

where

log^T^*K* is the protonation constant at infinite dilution (*I* → 0 mol dm^−3^);
${z^{*}=\Sigma \;(\mathrm{charge})}^{2}\mathrm{reactants}-{\Sigma \;(\mathrm{charge})}^{2}\mathrm{products}$;
$\mathrm{DH}=0.51\cdot (I^{0.5}/(1+1.5\cdot I^{0.5}))$ is the Debye–Hückel term;
*C* is an empirical parameter for the dependence of the protonation constants on the *I*/mol dm^−3^.

The assessment of the log^T^*K* value and *C* parameter is crucial for the calculation of equilibrium constants at any desired ionic strength condition within the experimental variable range under investigation, including those simulating the conditions of environmentally relevant fluids.

### 3.5. Calculations

The BSTAC computer programme [76] was employed for determining acid–base titration parameters, such as the standard electrode potential (*E*^0^), water ionic product (log*K*_w_) and acidic junction potential (*j*_a_), as well as for establishing the reagents’ analytical concentrations. Additionally, this programme was used for processing potentiometric data to calculate the protonation constants at various ionic strength conditions and at infinite dilution.

## 4. Conclusions

This study employed a multi-analytical approach to comprehensively investigate the acid-base, thermal, and surface properties of agri-food waste biomasses, including original (*dry*) bergamot, grape and olive pomaces and pre-treated samples with deionised water and nitric acid solutions. By addressing these properties in a single, detailed investigation, the research provides a robust foundation for developing efficient and sustainable strategies for the sequestration of metal cations from natural waters, contributing to the broader goals of environmental remediation and resource conservation.

The acid-base properties were explored at *t* = 25 °C across varying ionic strengths (*I* = 0.10–1.00 mol dm^−3^ in NaNO_3(aq)_) using different experimental approaches. Unlike previous studies, which often ignored ionic media or which had experiments limited to a single ionic strength condition, this work applied an extended Debye–Hückel-type equation for data modelling. This approach allowed for the determination of thermodynamic parameters at infinite dilution and acidic constants at multiple ionic strengths, including those simulating environmentally relevant fluids. Carboxylic groups were identified as the predominant active sites in the pomaces, with their abundance varying across the samples. Bergamot pomace pre-treated with water showed the highest concentration of -COOH groups, surpassing that of the corresponding grape and olive pomaces as well as that of other agri-foods and natural biomasses reported in the literature. Point of zero charge (PZC) analysis for BP H_2_O, GP H_2_O, and *dry* OP samples confirmed pH values at which the surface charge becomes neutral, aligning well with literature data. Surface area analysis via the BET method revealed values in the range of 0.50–0.75 m^2^ g^−1^, consistent with reported values for citrus peels and pomaces. SEM imaging showed that the biomasses consisted of irregularly shaped particles with varying size distributions, with lengths up to approximately 100 µm. Thermal analysis by TG-IR and DSC demonstrated that grape pomaces exhibited the highest thermal stability, while olive pomaces underwent the greatest mass loss. Overall, this comprehensive characterisation highlights the potential of these bio-based materials for environmental applications, particularly in the remediation of cationic pollutants. These findings advance the understanding of agri-food waste valorisation and lay the groundwork for future strategies in sustainable waste management and water treatment technologies.

## Figures and Tables

**Table 1:** Protonation constants, empirical *C* parameter (Equation (4)) and active site concentration (meq g^−1^) of BP, GP and OP determined at infinite dilution in NaNO_3(aq)_, at *t* = 25 °C.

Pre-Treatment	Species	log^T^*K*^H (a)^	*C*	meq g^−1^
H_2_O	H(BP)	3.65 ± 0.01 ^(b)^	−0.08 ± 0.02 ^(b)^	1.10 ± 0.02 ^(b)^
-	H(GP)	4.13 ± 0.03	−0.20 ± 0.04	0.85 ± 0.01
H_2_O	H(GP)	4.30 ± 0.03	−0.24 ± 0.04	0.23 ± 0.01
HNO_3_	H(GP)	4.69 ± 0.05	−0.44 ± 0.07	0.16 ± 0.01
-	H(OP)	4.29 ± 0.06	0.33 ± 0.13	0.43 ± 0.01
H_2_O	H(OP)	5.54 ± 0.08	−1.11 ± 0.12	0.21 ± 0.01
HNO_3_	H(OP)	4.52 ± 0.05	0.57 ± 0.08	0.15 ± 0.01

^(a)^ log^T^*K*^H^ refer to the equilibria H^+^ + BP^−^ = H(BP), H^+^ + GP^−^ = H(GP), H^+^ + OP^−^ = H(OP) for bergamot, grape, and olive pomaces, respectively; ^(b)^ ±Std. dev.

**Table 2:** Surface areas (m^2^ g^−1^) of the adsorbent materials obtained by BET analysis.

Sample/Pre-Treament	*Dry*	H_2_O	HNO_3_
BP	0.37 ^(a)^	0.62 ^(a)^	0.65 ^(a)^
GP	0.33	0.50	0.30
OP	0.53	0.75	0.58

^(a)^ ±std. dev. = 0.01–0.02.

**Table 3:** TGA data obtained for BP, OP and GP samples.

Sample	Step	Temperature Interval (°C)	Weight Loss (%)	Residual Weight at 800 °C (%)
*dry* BP	1	30–120	1.7	25
	2	120–210	12.0	
	3	210–300	24.0	
	4	300–410	25.0	
	5	410–600	10.0	
BP H_2_O	1	30–190	4.7	24
	2	190–300	28.7	
	3	300–410	30.0	
	4	410–610	9.8	
BP HNO_3_	1	30–190	4.9	20
	2	190–300	23.5	
	3	300–410	38.0	
	4	410–560	8.9	
*dry* OP	1	30–165	3.9	23
	2	165–265	10.5	
	3	265–310	17.0	
	4	310–390	29.0	
	5	390–550	12.6	
OP H_2_O	1	30–155	1.8	15.7
	2	155–265	11.0	
	3	265–320	20.0	
	4	320–410	36.5	
	5	410–550	10.0	
OP HNO_3_	1	30–150	1.6	16
	2	150–280	17.0	
	3	280–330	18.5	
	4	330–420	34.0	
	5	420–560	8.8	
*dry* GP	1	30–150	3.0	27.7
	2	150–250	8.7	
	3	250–295	9.5	
	4	295–355	18.7	
	5	355–420	16.4	
	6	420–600	12.0	
GP H_2_O	1	30–175	3.4	26
	2	175–275	7.0	
	3	275–325	12.0	
	4	325–415	31.0	
	5	415–570	14.6	
GP HNO_3_	1	30–180	3.0	27
	2	180–275	7.0	
	3	275–310	7.8	
	4	310–365	18.0	
	5	365–420	16.0	
	6	420–570	14.0	

## Data Availability

The original contributions presented in this study are included in the article/Supplementary Material. Further inquiries can be directed to the corresponding authors.

## Supplementary Materials

The following supporting information can be downloaded at: https://www.mdpi.com/article/10.3390/molecules29235735/s1, Table S1. Pre-treatments performed on *dry* bergamot (BP), grape (GP) and olive (OP) pomaces. Table S2. Acidic constants calculated at *t* = 25 °C and different ionic strengths in NaNO_3(aq)_ for BP, GP and OP species. Table S3. Chemicals used to perform the investigations, sourced from Merck (Darmstadt, Germany). Figure S1. ATR FT-IR spectra recorded for *dry* GP (a) and grape pomace pre-treated with nitric acid (b); *dry* OP (c) and olive pomace after pre-treatment with HNO_3_ (d). Figure S2. ΔpH vs. pH_i_ plots for the PZC determination for the GP H2O (a) and *dry* OP (b) samples. Figure S3. Superimposition of TGA thermograms of OP samples. Figure S4. Superimposition of TGA thermograms of GP samples. Figure S5. FT-IR of gases evolved during the decomposition of OP samples. The temperatures where the spectra were recorded are also reported: CO2 (black stars), water (red stars), formaldehyde (green stars), and CO (magenta stars). Figure S6. FT-IR of gases evolved during the decomposition of GP samples. The temperatures where the spectra were recorded are also reported. CO_2_ (black stars), water (red stars), formaldehyde (green stars), and CO (magenta stars). Figure S7. Superimposition of DSC curves of BP samples. Figure S8. Superimposition of DSC curves of OP samples. Figure S9. Superimposition of DSC curves of GP samples.
