# Supplementary material for: Multi-Analytical Approach for the Acid-Base, Thermal and Surface Properties Assessment of Waste Biomasses
Source: Molecules. 2024 Dec 5;29(23):5735. doi: 10.3390/molecules29235735 (PMC11643424; doi:10.3390/molecules29235735)
Supplement: Supplementary file 1 [file molecules-29-05735-s001.zip › molecules-3330996-supplementary.pdf]

Supplementary Material

Multi-analytical approach for the acid-base, thermal and surface properties assessment of waste biomasses

Salvatore Giovanni Michele Raccuia[1], Emanuele Zanda[1], Clemente Bretti[1], Mauro Formica[2], Eleonora Macedi[2], Andrea Melchior[3], Marilena Tolazzi[3], Martina Sanadar[3], Davide Lascari[4], Giovanna De Luca[1], Anna Irto[1,*], Concetta De Stefano[1], Paola Cardiano[1,*], Gabriele Lando[1]

[1] Dipartimento di Scienze Chimiche, Biologiche, Farmaceutiche e Ambientali, Università degli Studi di Messina, 98168, Messina, Italy; saraccuia@unime.it (S.G.M.R.), emanuele.zanda@unime.it (E.Z.); cbretti@unime.it (C.B.); giovanna-deluca@unime.it (G.D.L.); airto@unime.it (A.I.); cdestefano@unime.it (C.D.S.); pcardiano@unime.it (P.C.); glando@unime.it (G.L.).

[2] Dipartimento di Scienze Pure e Applicate, Università degli Studi di Urbino "Carlo Bo", 61029, Urbino, Italy; mauro.formica@uniurb.it (M.F.); eleonora.macedi@uniurb.it (E.M.).

[3] Dipartimento Politecnico di Ingegneria e Architettura, Laboratorio di Tecnologie Chimiche, Università di Udine, 33100, Udine, Italy; andrea.melchior@uniud.it (A.M.); marilena.tolazzi@uniud.it (M.T.); martina.sanadar@uniud.it (M.S.).

[4] Dipartimento di Fisica e Chimica– Emilio Segré, Università di Palermo, I-90128 Palermo, Italy; davide.lascari@unipa.it (D.L.).

*Corresponding authors: pcardiano@unime.it (P.C.); airto@unime.it (A.I.).

Table S1. Pre-treatments performed on *dry* bergamot (BP), grape (GP) and olive (OP) pomaces.

Cod.	Pomace amount (g)	Pretreatment	Yield (%)
BP H_2O	20.12603	200 cm^3 deionized H_2O	48
BP HNO_3	20.01570	200 cm^3 HNO_3 0.1019 mol dm^{-3}	40
GP H_2O	20.0862	200 cm^3 deionized H_2O	61
GP HNO_3	20.0975	200 cm^3 HNO_3 0.1019 mol dm^{-3}	62
OP H_2O	20.25491	200 cm^3 deionized H_2O	76
OP HNO_3	19.87594	200 cm^3 HNO_3 0.1019 mol dm^{-3}	79

Table S2. Acidic constants calculated at t = 25°C and different ionic strengths in $NaNO_{3(aq)}$ for BP, GP and OP species

Sample	Species	I/mol dm^{-3}				
		0.10	0.25	0.50	0.75	1.00
BP H_2O	H(BP)	3.43	3.34	3.26	3.21	3.16
dry GP	H(GP)	3.90	3.79	3.69	3.60	3.53
GP H_2O	H(GP)	4.30	4.28	4.32	4.38	4.52
GP HNO_3	H(GP)	4.42	4.28	4.11	3.97	3.84
dry OP	H(OP)	4.11	4.12	4.19	4.28	4.39
OP H_2O	H(OP)	5.20	4.97	4.63	4.32	4.01
OP HNO_3	H(OP)	4.35	4.36	4.43	4.53	4.63

Table S3. Chemicals used to perform the investigations, sourced from Merck (Darmstadt, Germany)

Chemical	Formula	CAS n°	Assay (mass)
Nitric acid	HNO_3	7697-37-2	≥ 99%
Sodium hydroxide	NaOH	1310-73-2	≥ 99%
Potassium phthalate monobasic	$C_8H_5O_4K$	877-24-7	≥ 99.95%
Sodium carbonate	Na_2CO_3	497-19-8	99.995%
Sodium nitrate	$NaNO_3$	7631-99-4	≥ 99%
Calcium acetate hydrate	$C_3H_6CO_4Ca \cdot xH_2O$	114460-21-8	≥ 99%

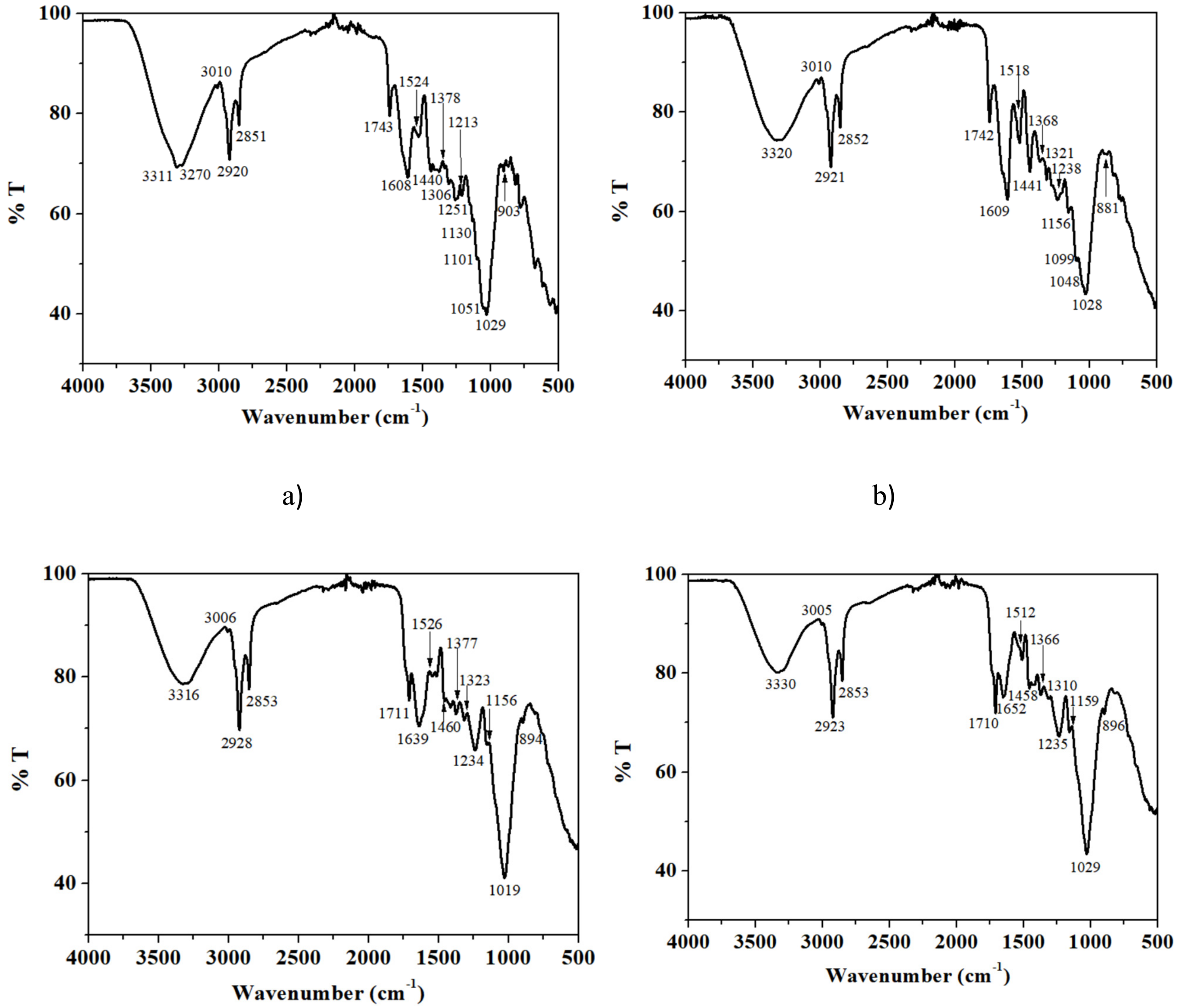

c)

d)

Figure S1. ATR FT-IR spectra recorded for *dry* GP (a) and grape pomace pre-treated with nitric acid (b); *dry* OP (c) and olive pomace after pre-treatment with HNO_3 (d).

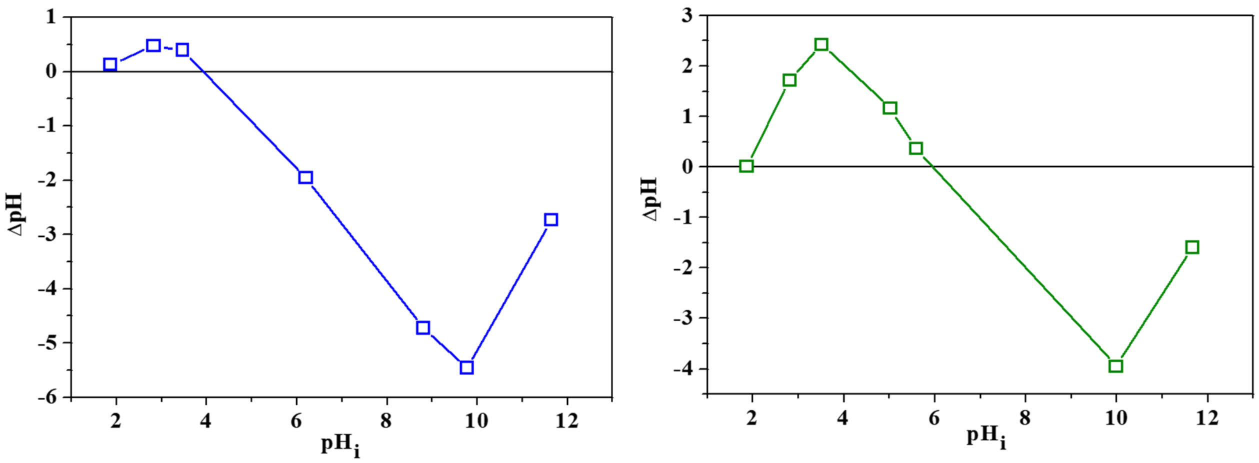

a) b)

Figure S2. ΔpH *vs*. pH$_i$ plots for the PZC determination for the GP H_2O (a) and *dry* OP (b) samples.

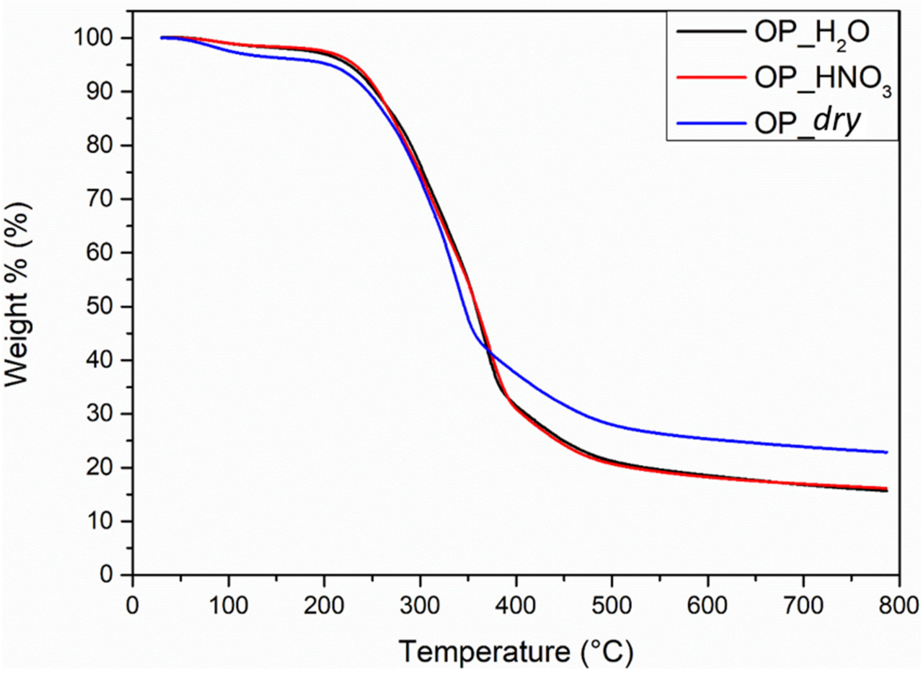

Figure S3. Superimposition of TGA thermograms of OP samples.

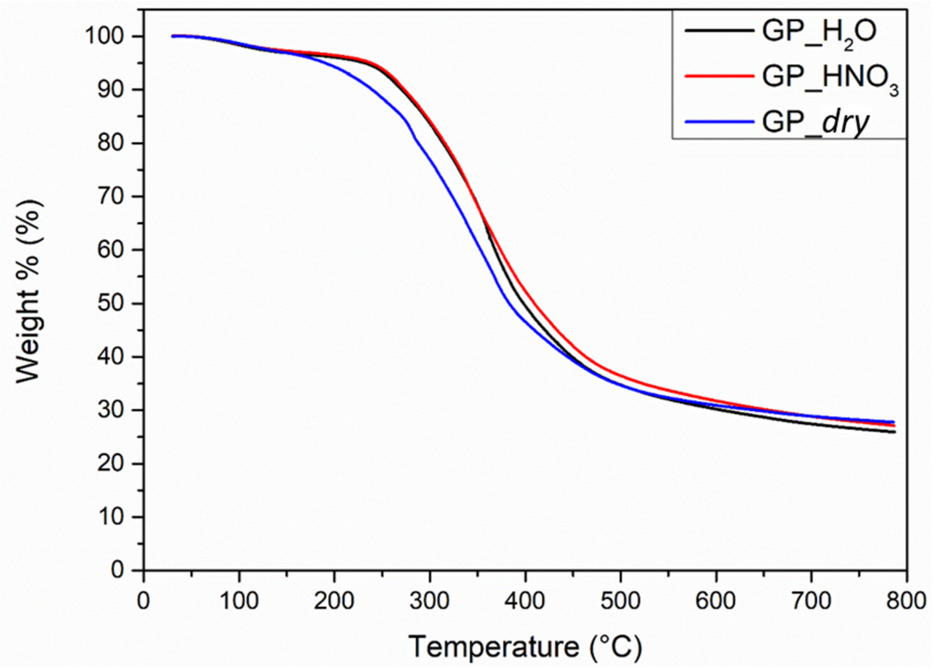

Figure S4. Superimposition of TGA thermograms of GP samples.

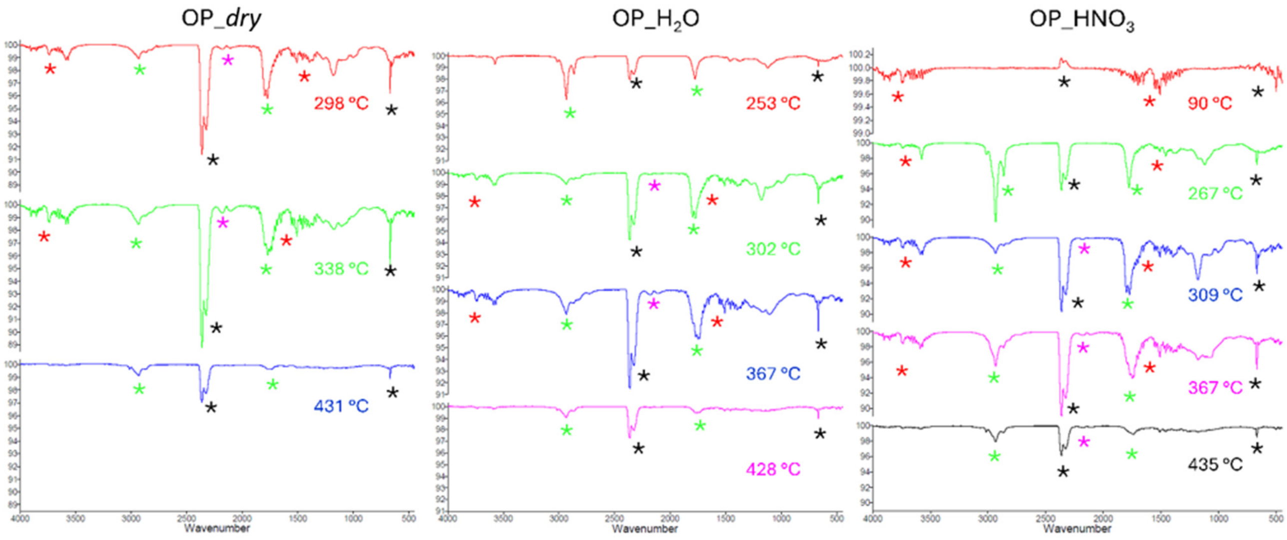

Figure S5. FT-IR of gases evolved during the decomposition of OP samples. The temperatures where the spectra were recorded are also reported: CO_2 (black stars), water (red stars), formaldehyde (green stars), and CO (magenta stars).

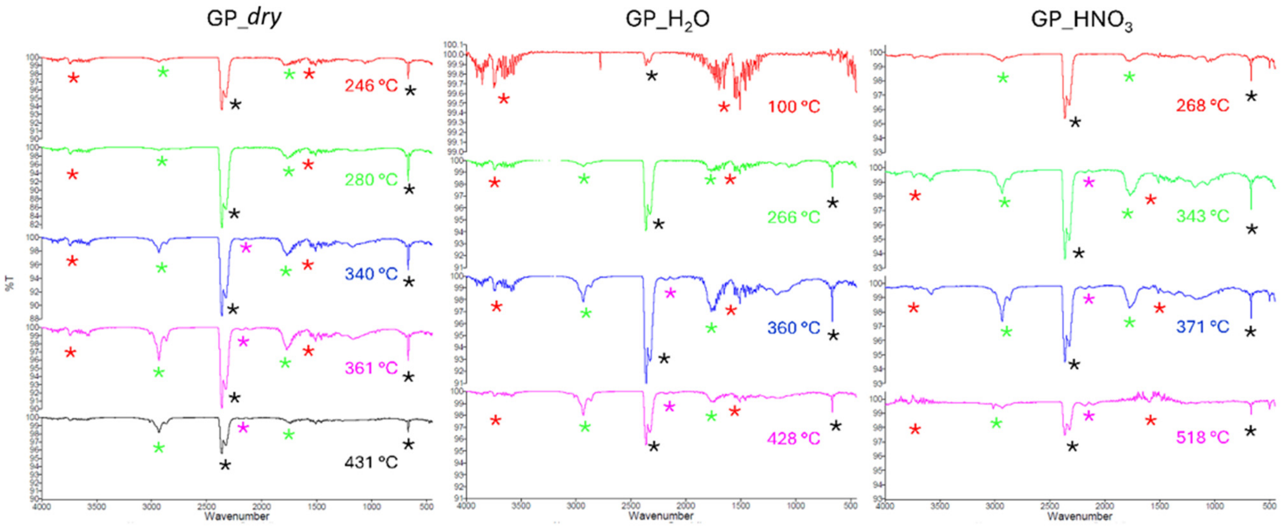

Figure S6. FT-IR of gases evolved during the decomposition of GP samples. The temperatures where the spectra were recorded are also reported. CO_2 (black stars), water (red stars), formaldehyde (green stars), and CO (magenta stars).

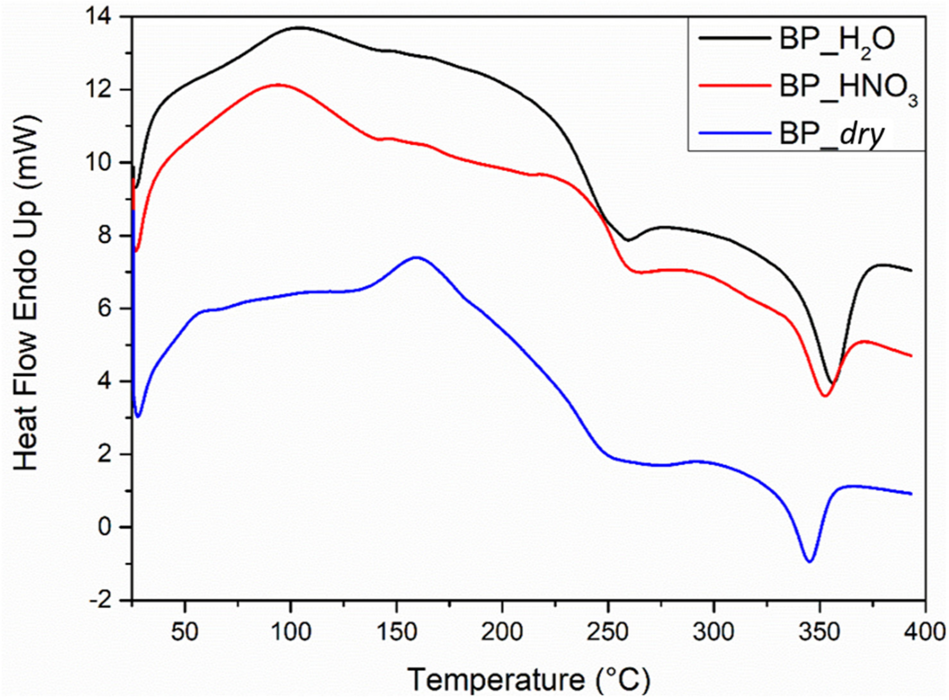

Figure S7. Superimposition of DSC curves of BP samples.

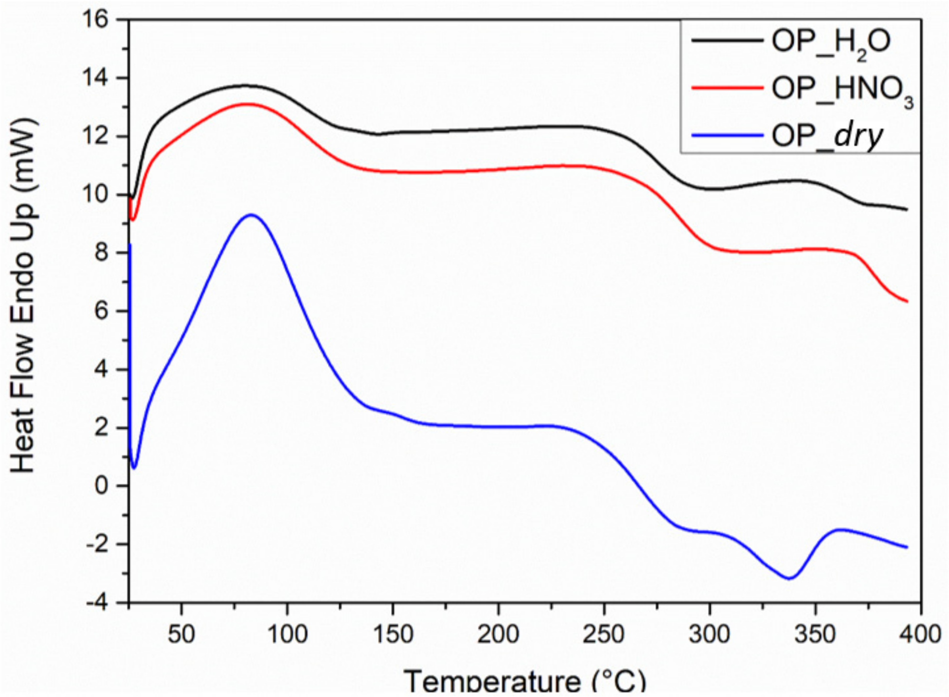

Figure S8. Superimposition of DSC curves of OP samples.

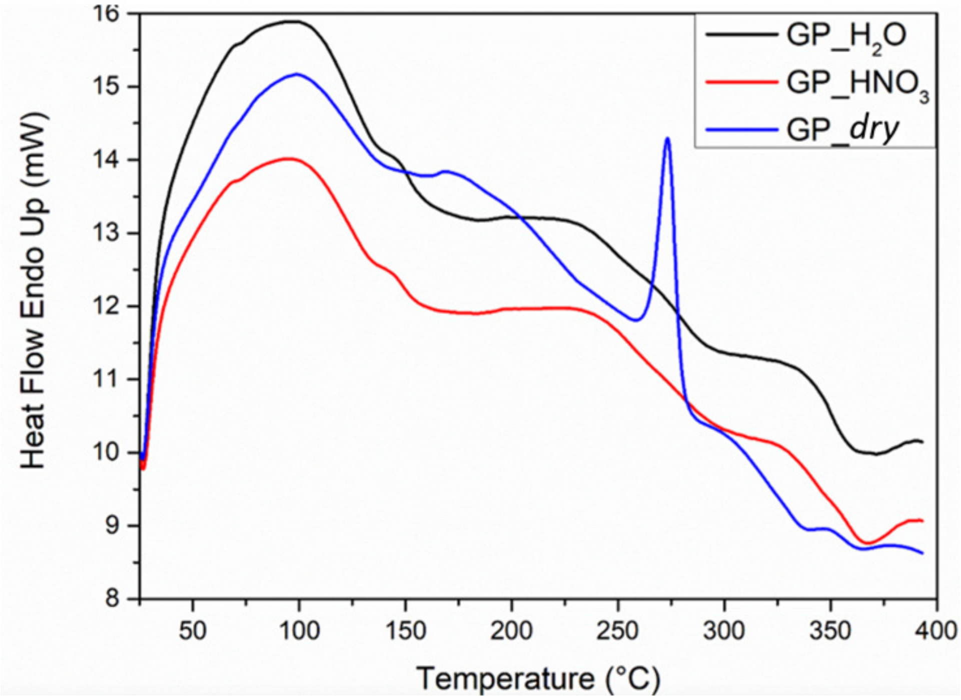

Figure S9. Superimposition of DSC curves of GP samples.
